# Influence of Extraction Conditions on Organic Carbon Recovery from Biogas Digestate

**DOI:** 10.1021/acsomega.6c04180

**Published:** 2026-09-23

**Authors:** Eglė Didžiulytė, Rasa Šlinkšienė

**Affiliations:** Kaunas University of Technology, Kaunas 50254 Lithuania

## Abstract

Intensive use of mineral fertilizers has reduced natural soil fertility and increased the demand for sustainable nutrient sources. In this study, three digestates obtained from Lithuanian biogas plants were chemically and instrumentally characterized and evaluated as potential sources of organic carbon and plant nutrients. The dried digestates contained 52.9–59.5% organic carbon and appreciable amounts of nitrogen, phosphorus, and potassium, although the measured nutrient concentrations depended on the applied extraction or mineralization procedure. The influence of key extraction parameters, including extractant type (water or 0.5 M KOH), temperature, extraction time, particle size, and extraction method (magnetic stirring or ultrasonication), on organic carbon recovery was systematically investigated. Alkaline extraction markedly increased TOC concentrations in the extracts, yielding values up to approximately 8–29 times higher than those obtained by aqueous extraction after 3 days. The highest TOC concentration (4495 mg/L) was obtained for KP digestate extracted with 0.5 M KOH at 90 °C using magnetic stirring for 6 h. In aqueous extracts, the maximum TOC concentration reached 715 mg/L under ultrasonication at 50 °C. Temperature, extraction time, and particle size had condition- and digestate-dependent effects on TOC recovery. The ultrasonic extraction series yielded the highest TOC concentration among the aqueous extracts, whereas the magnetic-stirring series yielded the highest value under alkaline conditions. In addition, carbon recovery efficiency was calculated relative to the initial organic carbon content of the digestates, providing a quantitative assessment of carbon transfer from the solid material to the liquid phase. The highest carbon recovery efficiency reached approximately 76%. These results identify the extraction conditions that enhance soluble organic carbon recovery from digestate and provide a basis for further chemical characterization and application-oriented evaluation of the obtained extracts.

## Graphical abstract

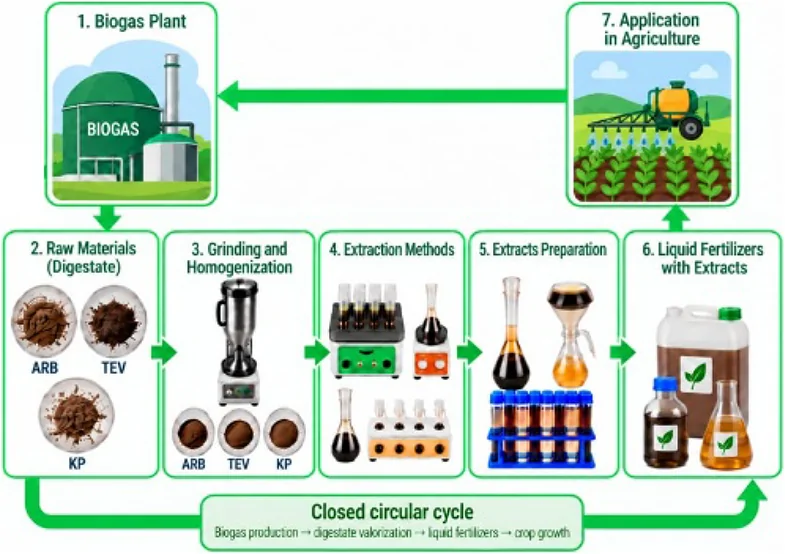

## 1 Introduction

Soil organic carbon is essential for maintaining the soil structure, water retention, nutrient supply, biological activity, and resilience to environmental disturbances. Agricultural soils may lose organic carbon as a result of intensive cultivation, erosion, climate-related processes, and an insufficient return of organic residues. Therefore, the recovery and reuse of organic carbon from residual biomass may contribute to circular soil management strategies. However, the practical value of recovered carbon depends on its chemical composition, stability, and behavior after soil application.^1,2^

Against this background, the expansion of full-scale anaerobic digestion for renewable energy production and organic-waste treatment is increasing the quantities of digestate that require effective management and valorization.^3,4^ Digestate, a byproduct of the anaerobic digestion of organic waste, contains plant nutrients and organic carbon, although its composition varies considerably depending on the feedstock and digestion process.^4–6^ Although digestate is commonly applied directly to agricultural soils, its high moisture content, heterogeneous composition, and variable nutrient availability may complicate storage and transportation and limit predictable nutrient use.^4–7^ Solid–liquid separation is therefore frequently applied as an initial processing step, generating solid and liquid fractions that can be handled and valorized separately.^7,8^ Subsequent recovery processes may further concentrate selected nutrient or organic components and facilitate their targeted utilization.^4,7,9^

Digestate-derived organic fractions, including humic and humic-like substances, have been investigated within broader nutrient-recovery and digestate-valorization frameworks. Drosg et al. reviewed technological approaches for nutrient recovery from biogas digestate,^7^ while Lehto and Järvelä proposed an integrated cascading process combining phosphorus and nitrogen recovery with ammonia-assisted extraction of organic substances from anaerobic digestate.^9^ Guilayn et al. extracted humic-like substances from sewage-sludge and manure digestates and evaluated their biostimulatory effects in hydroponic lettuce cultivation.^10^ Nayak and Ranade further summarized digestate characteristics, potential valorization products, available processing technologies, and associated challenges.^4^ Han et al. optimized KOH-assisted hydrothermal treatment of corn-straw digestate by varying temperature, reaction time, and KOH dosage.^11^ Anielak et al. recovered humic substances from anaerobically digested municipal sewage sludge using alkaline dissolution, centrifugation, and subsequent product separation,^12^ while Xiao et al. reviewed the quantification, recovery efficiency, and methodological limitations associated with humic-substance recovery from anaerobically digested sludge.^13^

Taken together, these studies demonstrate the potential for recovering organic fractions from digestate but generally focus on a specific feedstock, one selected treatment route, or subsequent characterization and agronomic evaluation of the recovered material. Direct systematic comparisons of aqueous and alkaline extractions across several compositionally distinct solid digestates obtained from full-scale biogas plants remain scarce. In particular, the effects of the extraction medium, temperature, extraction time, particle size, and extraction mode have rarely been examined together within a common comparative framework. The novelty of the present study lies in the comparative evaluation of these extraction factors across three chemically and structurally distinct solid digestates by using the same experimental approach. This comparison also enabled different effective processing routes to be identified: alkaline extraction assisted by heating and magnetic stirring and aqueous extraction enhanced by ultrasonication. Preliminary results from the ultrasonic bath extraction series were previously presented in a conference contribution.^14^ The present study substantially extends that preliminary work by integrating static, temperature-controlled, magnetic stirring, and ultrasonic extraction experiments with comprehensive chemical and instrumental characterization, statistical evaluation, and carbon recovery calculations.

Organic carbon is an important component of solid digestate, and its amount and composition depend strongly on the original feedstock and anaerobic digestion process.^4,5,15^ Digestate-derived organic matter therefore represents a potential target for further valorization, as demonstrated by previous recovery studies.^9–13^ In extraction studies, the total organic carbon concentration in the liquid phase provides a quantitative indicator of extraction performance, while carbon recovery efficiency expresses the proportion of the initial organic carbon content of the digestate that is transferred from the solid material to the extract. However, these indicators do not by themselves describe the chemical composition, long-term stability, or agronomic value of recovered organic compounds.

Therefore, this study aimed to determine the influence of the extraction medium, temperature, extraction time, particle size, and extraction mode on organic carbon recovery from the dried solid fractions of three digestates obtained from Lithuanian biogas plants. The digestates were chemically, structurally, and thermally characterized, and total organic carbon concentrations and carbon recovery efficiencies were determined under different aqueous and alkaline extraction conditions by using magnetic stirring and ultrasonication. This integrated approach enabled the extraction behavior to be related to differences in digestate composition and allowed the dominant factors controlling organic carbon transfer into the liquid phase to be identified and compared among the three digestates.

## 2 Materials and Methods

### 2.1 Raw Materials

Three mechanically separated solid digestate fractions, obtained as byproducts of anaerobic digestion, were collected from three full-scale biogas plants in Lithuania: UAB “AGARAS” (ARB), located in Biržai municipality; UAB “Tvari energija” (TEV), located in Vievis municipality; and UAB “Kurana” (KP), located in Pasvalys municipality. The feedstocks used for anaerobic digestion differed among the biogas plants: TEV: expired food products, substandard products, oils, slaughterhouse, and restaurant waste; ARB: slaughterhouse waste (blood, intestines), manure, straw, unsuitable feed, and other green mass; KP: nonfood grain residues and food production waste. In all cases, the mechanically separated wet solid fraction of the digestate was used. To reduce moisture content and facilitate storage, the samples were dried at 60 °C to constant mass. Representative images of the wet and dried digestates are shown in Figure 1.

**Figure 1:**
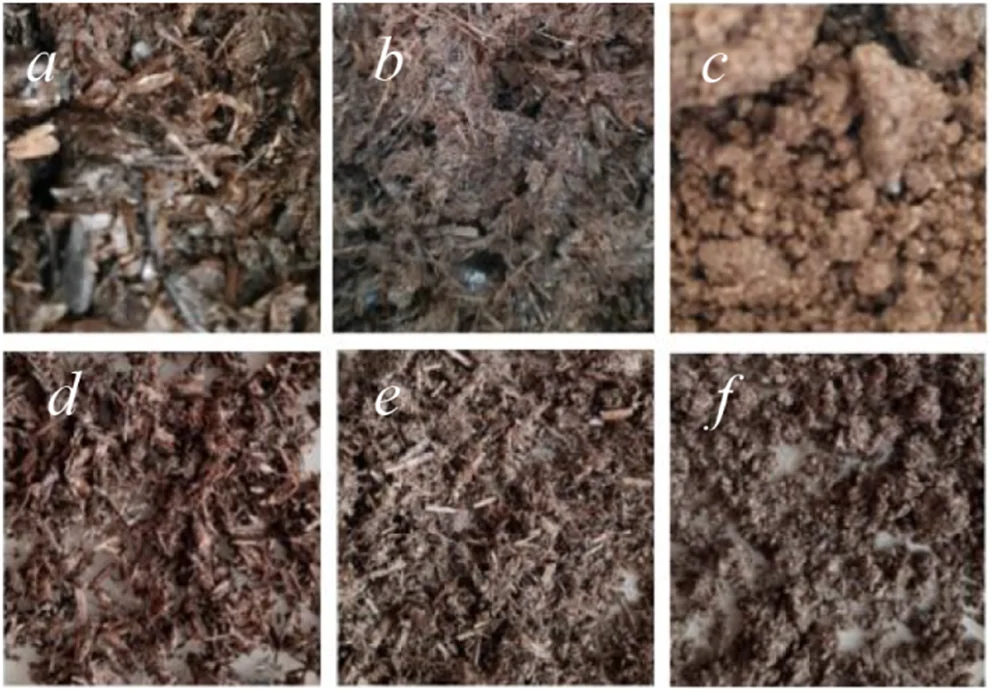
Representative images of the digestates: a, d: ARB; b, e: TEV; c, f: KP; a, b, c: wet samples; d, e, f: dried samples.

Dried digestates were sieved and fractionated to evaluate the influence of particle size on extraction efficiency. Two particle size fractions were selected for further experiments: whole digestate (WD) with a particle size of 3–5 mm and crushed digestate (CD) with a particle size <2 mm. Crushing was performed using a Waring Two Speed Blender 8011S (240 VAC, 50–60 Hz, 3.6 A; Connecticut, USA), and particle size separation was achieved by sieving. Unless otherwise specified, the dried digestate samples were used for chemical and instrumental analyses.

Distilled water and the analytical-grade reagents, supplied by UAB “EuroChemicals” were used in the study: hydrochloric acid (HCl, 37%), sulfuric acid (H_2_SO_4_, 98%), calcium chloride (CaCl_2_, ≥99%), potassium hydroxide (KOH, ≥85%), potassium dichromate (K_2_Cr_2_O_7_, ≥99.5%), d-glucose (≥99.5%), disodium ethylenediaminetetraacetate (EDTA; Trilon B, ≥99%), ammonium nitrate (NH_4_NO_3_, ≥98%), ammonia solution (NH_4_OH, 25%), and Devarda’s alloy. The reagents were used without further purification.

### 2.2 Extraction Procedure

Extraction experiments were performed at a solid-to-liquid ratio of 1:50 (w/v) using 1.00 g dry digestate per 50 mL of extractant. Distilled water and 0.5 M KOH were used as extractants. Each experimental condition was tested using three independently prepared extracts. The extraction experiments were carried out under the following conditions: static extraction at 23 °C for 3, 6, and 9 days; magnetic stirring at 23, 50, 70, and 90 °C for 3, 6, and 9 h; ultrasonic-bath extraction at 30, 50, and 60 °C for 3, 6, and 9 h; and temperature-controlled extraction without additional mixing at 50, 70, and 90 °C for 3, 6, and 9 h. Mixing was applied either by stirring using IBX Instruments HO 1 Series STIH-001-001 magnetic stirrers (power output: 180 W, stirring speed range: 1000 rpm) or 3 L Ultrasonic Cleaner Proclean 3.0 ECO ultrasonic bath operated at a nominal frequency of 40 kHz and a nominal ultrasonic power of 80 W (ultrasonic power supply: 230 V, 50 Hz). Samples were treated for 3, 6, or 9 h using continuous operation throughout the extraction process. All extraction experiments were performed for the three digestates and both particle size fractions using both extraction media. After extraction, the suspensions were filtered, and the liquid phase was used for total organic carbon (TOC) concentration determination. Prior to TOC determination, all filtrates were quantitatively diluted to a final 100 mL. Carbon recovery calculations were performed on the final solution volume (0.100 L), thereby accounting for the applied dilution factor.

### 2.3 Chemical Analysis

For the determination of water- and acid-soluble inorganic nitrogen forms, 1.0 g of dried digestate was mixed with 50 mL of distilled water or HCl solution prepared by mixing concentrated HCl and water at a ratio of 1:1 (v/v). The suspensions were kept at room temperature for 24 h and subsequently filtered. Ammonium nitrogen (N-NH_4_^+^) was determined in the filtrates using automated distillation (VAPODEST 45s, Gerhardt, Germany). For nitrate nitrogen (N-NO_3_^–^) determination, nitrate was reduced to ammonia using Devarda’s alloy prior to distillation. The sum of the determined inorganic nitrogen forms (N_sum_) was calculated as the sum of N-NH_4_^+^ and N-NO_3_^–^. Total nitrogen (N_total_) was determined according to EN 16169:2012 ^16^ after digestion of the dried digestate samples with concentrated H_2_SO_4_ in the presence of Kjeldahl catalyst tablets, followed by automated distillation. All nitrogen results were recalculated and expressed as percentage on a dry-matter basis.

The phosphorus concentration in the aqueous and HCl media was determined spectrophotometrically using the procedure described in ISO 6878:2004.^17^ Absorbance was measured at 450 nm using a UV–vis spectrophotometer (T70+, PG Instruments, UK). The measured phosphorus concentrations were converted to P_2_O_5_ using the conversion factor of 2.291, in accordance with conventional fertilizer reporting practices.^18^

The potassium concentration in the aqueous and HCl media was determined by flame photometry^19^ using a PFP7 flame photometer (Jenway, UK). The measured potassium concentrations were converted to K_2_O using a conversion factor of 1.205.^18^

Calcium and magnesium concentrations in the aqueous and HCl media were determined by complexometric titration with EDTA using the procedure described in LST EN 16198:2013. The sum of calcium and magnesium was determined first, calcium was determined separately, and magnesium was calculated from the difference between the two titration results.^20^ The results were converted and expressed as CaO and MgO, respectively.^18^

Organic matter content was determined gravimetrically in a muffle furnace at 900 °C for 30 min (loss on ignition), then cooling and weighing the remaining residue. The residual mass was considered as ash, and organic matter content was calculated as the difference between the initial dry sample mass and the ash mass.^21^ Organic carbon (OC) content in the dried digestate samples was determined using the sulfochromic oxidation procedure described in ISO 14235:1998.^22^ Total organic carbon (TOC) concentrations in liquid extracts were measured using a TOC analyzer (TOC-L, Shimadzu, Japan) in accordance with LST EN 1484:2002.^23^ When necessary, the liquid extracts were diluted prior to analysis to ensure that the measured carbon concentrations were within the analytical working range. Total organic carbon in the liquid extracts was determined in nonpurgeable organic carbon (NPOC) mode. Before combustion analysis, the samples were acidified to pH < 2 and sparged with carrier gas to remove inorganic carbon present as dissolved carbonate and bicarbonate.

Trace elements and heavy metal (Fe, Mn, Cu, Cr, Zn, Co, Ni, Pb, and Cd) concentrations after digestate sample treatment with water and diluted hydrochloric acid were analyzed by atomic absorption spectroscopy (Analyst 400, PerkinElmer, USA). The AAS results obtained in mg/L were recalculated to mg per gram of dry digestate, taking into account the initial sample mass of 1.00 g, the final solution volume of 250 mL, and any applied dilution factors.

### 2.4 Instrumental Analysis

Fourier transform infrared (FT-IR) spectra were recorded using a Bruker Vertex 70 spectrometer (Bruker Optics GmbH & Co. KG, Ettlingen, Germany) over the range of 4000–400 cm^–1^ at a resolution of 1 cm^–1^. The samples were prepared using the KBr pellet method, and the obtained spectra were interpreted by comparison with reference data reported in the relevant spectral handbook by Nakamoto.^24^

X-ray diffraction (XRD) analysis was performed using a Bruker D8 Advance diffractometer (Bruker AXS KG, Bremen, Germany) with CuKα radiation and a nickel (Ni) filter. Diffraction patterns were collected over a 2θ range of 5–70°. The measurements were carried out at an anodic voltage of 40 kV and a current of 40 mA. The detector step size was set to 0.02°, with an intensity acquisition time of 0.2 s per step. Phase identification was performed using Crystallographica Search-Match software (version 2.1.1.1) by comparing the obtained diffraction peaks with reference diffraction patterns from the Search-Match database.

Simultaneous thermal analysis (STA) was conducted using a LINSEIS STA PT-1000 thermal (Linseis Messgeraete GmbH, Selb, Germany) analyzer. Measurements were performed from 25 to 800 °C at a heating rate of 10 °C min^–1^ under a nitrogen atmosphere.^25^

Scanning electron microscopy (SEM) was carried out using an FEI Quanta 200 FEG microscope (Hitachi, Tokyo, Japan) at an accelerating voltage of 5 kV to evaluate digestate-particle morphology.^26^ Images were recorded at ×50 magnification.

### 2.5 Physicochemical Properties

The pH of the raw digestates was measured by using a digital pH meter (Hanna Instruments 221, Woonsocket, RI, USA). A digestate suspension was prepared at a solid-to-liquid ratio of 1:50 (w/v) using distilled water or a 0.01 M CaCl_2_ solution. The suspensions were equilibrated for 24 h at room temperature and filtered, and the pH was measured in the filtrates.

The moisture content of the wet and dried digestate samples was determined using an electronic moisture analyzer Kern MLS 50-3HA160N (KERN & Sohn, Balingen, Germany) at 100 °C until constant mass.

Electrical conductivity (EC) measurements were performed using a WTW inoLab Cond 720 conductivity meter (WTW, Weilheim, Germany). The EC of aqueous and potassium hydroxide (KOH) extracts prepared from the samples was determined after extraction and equilibration at room temperature. The obtained conductivity values were recorded and expressed in mS cm^–1^.

The particle size distribution of the raw materials was determined by using RETSCH woven wire sieves with mesh sizes of 0.2, 0.5, 1.0, 2.0, 3.15, 4.0, 5.0, 7.0, and 10.0 mm (Retsch, GmbH, Haan, Germany).

### 2.6 Carbon Recovery Calculations

The amount of organic carbon recovered in the liquid phase was calculated from the measured total organic carbon (TOC) concentration using a mass-balance approach previously applied for the quantification of extractable organic carbon.^27^ Each extraction was performed using 1.00 g of dry digestate and 50 mL of extractant, and the obtained filtrates were quantitatively diluted to a final volume of 0.100 L. Therefore, the mass of organic carbon recovered per gram of dry digestate was calculated as

$$m_{C,rec}=\frac{\mathrm{TOC}\times V}{1000\times m_{d}}$$

where *m*_C,rec_ is the amount of organic carbon recovered per gram of dry digestate, expressed as g C/g dry digestate; TOC is the organic carbon concentration in the final diluted extract, expressed in mg C/L; *V* is the final extract volume expressed in L; *m*_d_ is the mass of dry digestate used for extraction, expressed in g; and the factor 1000 converts milligrams to grams.

Carbon recovery efficiency was calculated relative to the initial organic carbon content of the dry digestate:

$$\eta_{C}=\frac{m_{C,rec}}{m_{C,init}}\times 100$$

where η_C_ is the carbon recovery efficiency, expressed as %, and *m*_C,init_ represents the initial amount of organic carbon per gram of dry digestate, calculated from the measured organic carbon content of the raw material and expressed as g C/g dry digestate.

The initial organic carbon content *m*_C,init_ was calculated as

$$m_{C,init}=\frac{{\mathrm{OC}}_{raw}}{100}$$

where *OC*_raw_ is the organic carbon content of the dried digestate determined by dichromate oxidation, expressed as % of dry matter. Because the calculations were normalized to 1 g of dry digestate, division by 100 directly converts the organic carbon content from % to g of C/g of dry digestate. For presentation in Table 3, the recovered carbon values were converted from g C/g to mg C/g dry digestate.

### 2.7 Statistical Analysis

All extraction experiments were performed in triplicate using independently prepared extracts, and TOC was determined separately in each extract. The results are presented as mean ± standard deviation (SD, *n* = 3). For the extraction experiments, statistical analysis was performed separately for each set of fixed extraction conditions presented in the individual panels of Figures 8–11. Within each panel, every unique combination of digestate type, particle size fraction, and extraction time was treated as a separate group. The groups were compared using one-way analysis of variance (ANOVA), followed by Tukey’s honestly significant difference (HSD) post hoc test. Differences were considered statistically significant at *p* < 0.05. Different lowercase letters above the bars indicate statistically significant differences among the means presented within the same panel. The chemical composition results presented in Figures 3 and 4 are expressed as mean ± SD (*n* = 3) and were evaluated descriptively without inferential statistical comparisons.

## 3 Results and Discussion

### 3.1 Physicochemical and Morphological Properties of Digestate

To characterize the digestate materials, their moisture content, particle size distribution, pH, and particle morphology were evaluated. The moisture content of the mechanically separated wet digestates was high and relatively similar, amounting to 73.7 ± 2.6% for ARB, 76.8 ± 0.2% for TEV, and 79.4 ± 0.3% for KP. The measured moisture contents were within the same numerical range as the 60–80% moisture range reported as favorable for anaerobic digestion.^28^ Nevertheless, the present values characterize the separated digestates after digestion and therefore should not be interpreted as evidence of optimal moisture conditions during the digestion process. High moisture content is a characteristic feature of digestate materials and affects their storage stability, transport, and subsequent processing. During storage prior to drying, visible mold growth was observed in the KP digestate, under both open and closed storage conditions, whereas no visible mold growth was observed in the ARB and TEV samples under the same conditions. Due to the high initial moisture content and difficult storage, all digestates were dried at 60 °C to constant mass before further analysis. After the drying process, the moisture contents of the digestate samples were: 8.2 ± 0.8% for ARB, 8.5 ± 0.7% for TEV, and 11.2 ± 0.6% for KP.

The granulometric composition of the dried samples (Table 1) demonstrated that the digestates were dominated by larger particle fractions (3–10 mm). Fractions ≥ 3 mm accounted for 74.3%, 68.6%, and 75.3% of the ARB, TEV, and KP samples, respectively, whereas particles <1 mm represented only 3.2–5.6% of the sample mass. Studies on plant biomass have shown that particle size reduction can increase extraction yield, although the effect depends on biomass composition and the extent of structural disruption rather than on particle size alone.^29^

**Table 1:** Particle Size Distribution of the Dried Digestates^a^

	Mass fraction of digestate particles, %		
Particle size, mm	ARB	TEV	KP
>10	–	17.8 ± 0.7	6.3 ± 0.2
7–10	27.2 ± 0.2	17.4 ± 0.6	14.4 ± 0.6
5–7	19.8 ± 0.1	13.9 ± 0.3	28.2 ± 0.6
3–5	27.3 ± 0.2	19.4 ± 0.1	26.5 ± 0.3
2–3	13.64 ± 0.02	13.6 ± 0.3	13.2 ± 0.4
1–2	8.9 ± 0.2	12.3 ± 0.2	8.2 ± 0.1
0.5–1	1.51 ± 0.01	2.6 ± 0.1	1.3 ± 0.1
0.2–0.5	1.6 ± 0.1	2.04 ± 0.03	1.35 ± 0.03
<0.2	0.07 ± 0.01	0.97 ± 0.01	0.7 ± 0.1

a Values are presented as mean ± SD (*n* = 3). “–” indicates that the fraction was not detected.

The pH values of 2% (w/v) aqueous suspensions were 7.3, 7.6, and 7.3 for ARB, TEV, and KP, respectively, indicating near-neutral conditions under the applied measurement conditions. When measured in 0.01 M CaCl_2_ solution, the pH values were 0.5–0.8 units lower than those measured in water, reaching 6.6, 6.8, and 6.5 for ARB, TEV, and KP, respectively.

SEM revealed that all digestate samples (Figure 2) exhibited aggregated structures with uneven and heterogeneous surfaces, reflecting the complex physical organization of digested biomass. At the same time, distinct morphological differences between the samples were observed.

**Figure 2:**
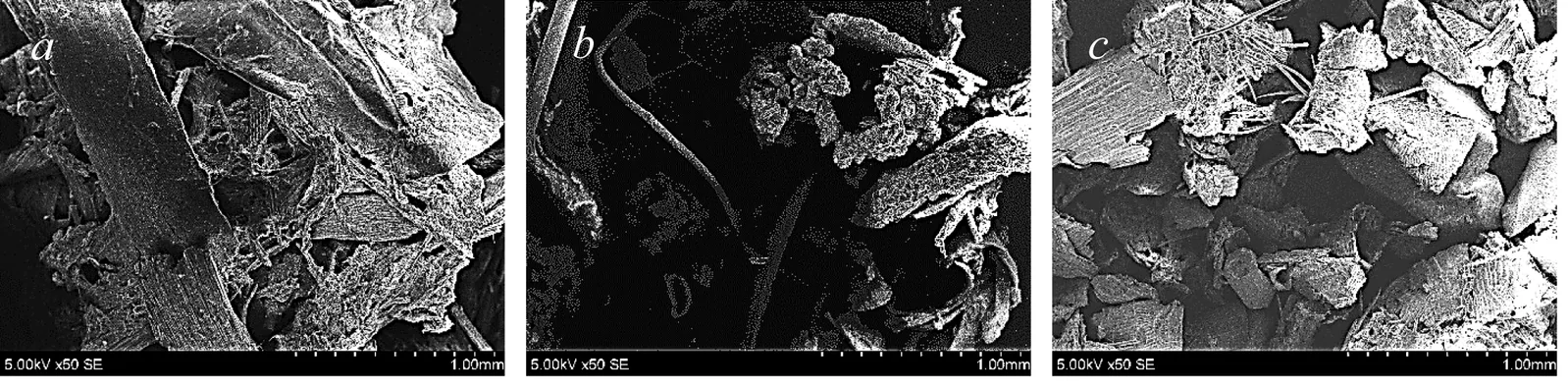
SEM images of dried digestate particles: a: ARB, b: TEV, and c: KP (magnification: ×50).

ARB and TEV digestates (Figure 2a,b) showed more pronounced fibrous features and elongated structural elements, suggesting the presence of residual fibrous organic matter and organic fibers. In contrast, the KP (Figure 2c) digestate consisted predominantly of irregular, compact particles of variable size and exhibited fewer visible fibrous structures. At the examined magnification (×50), the KP material also appeared more compact and contained fewer visible voids than the ARB and TEV samples. These observations are broadly consistent with the findings of Loffredo et al., who reported heterogeneous surfaces characterized by irregular ridges, channels, and cavities, together with generally low porosity, in solid digestate and its aerobically treated derivatives. The authors attributed this heterogeneity to the composition of the starting biomass and the biological conversion processes.^30^ Differences among the digestates examined in the present study may likewise reflect their distinct feedstocks and processing histories. Studies on other biomass matrices have shown that microstructural disruption can influence mass transfer and extraction efficiency; therefore, the morphological differences among the digestates may have contributed to their different extraction behavior.^29^

### 3.2 Chemical Analysis of Digestate

Digestate is generally considered a nutrient-rich material containing appreciable amounts of nitrogen.^6^ To characterize the inorganic nitrogen fraction released under different treatment conditions, ammonium nitrogen (N-NH_4_^+^), nitrate nitrogen (N-NO_3_^–^), and their calculated sum (N_sum_) were determined following treatment of the dried digestates with water or diluted HCl. The results are presented in Figure 3.

**Figure 3:**
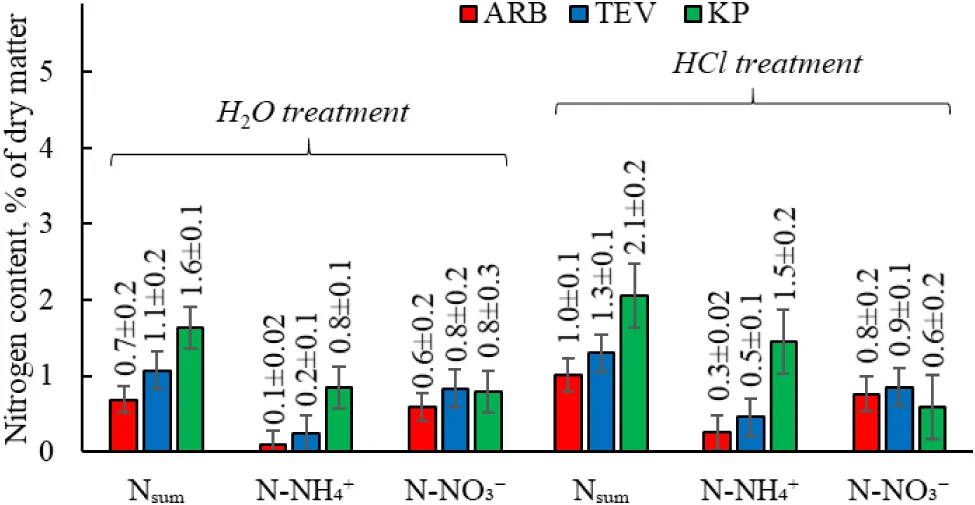
Contents of ammonium nitrogen, nitrate nitrogen, and their sum in dried digestates after treatment with water or diluted hydrochloric acid. Values are presented as mean ± SD (*n* = 3).

The sum of the determined inorganic nitrogen forms was highest in the KP digestate, reaching 1.6% after treatment with water and 2.1% after treatment with diluted HCl. ARB and TEV digestates exhibited lower values under both treatment conditions. The relative proportions of ammonium and nitrate nitrogen varied among the digestates. In KP, N-NH_4_^+^ and N-NO_3_^–^ contents were similar following water treatment (approximately 0.8% each), whereas ammonium nitrogen predominated after HCl treatment (approximately 1.5% compared with 0.6% nitrate nitrogen). In contrast, N-NO_3_^–^ predominated in both ARB and TEV digestates after treatment with either water or diluted HCl; after water treatment, N-NH_4_^+^ remained at or below 0.24%, whereas nitrate nitrogen was approximately 0.6–0.8%.

Total nitrogen determined after sulfuric acid mineralization was substantially higher than the sum of inorganic nitrogen forms determined after treatment with water or diluted HCl, reaching 3.2 ± 0.2% in ARB, 4.2 ± 0.5% in TEV, and 4.5 ± 0.3% in KP on a dry-matter basis.

The P_2_O_5_ and K_2_O contents determined following treatment of the dried digestates with water or diluted HCl are presented in Figure 4. The P_2_O_5_ contents were consistently higher following HCl treatment than following water treatment, indicating greater phosphorus solubilization under acidic conditions. KP showed the highest P_2_O_5_ content under both treatment conditions, TEV showed intermediate values, and ARB showed the lowest values.

**Figure 4:**
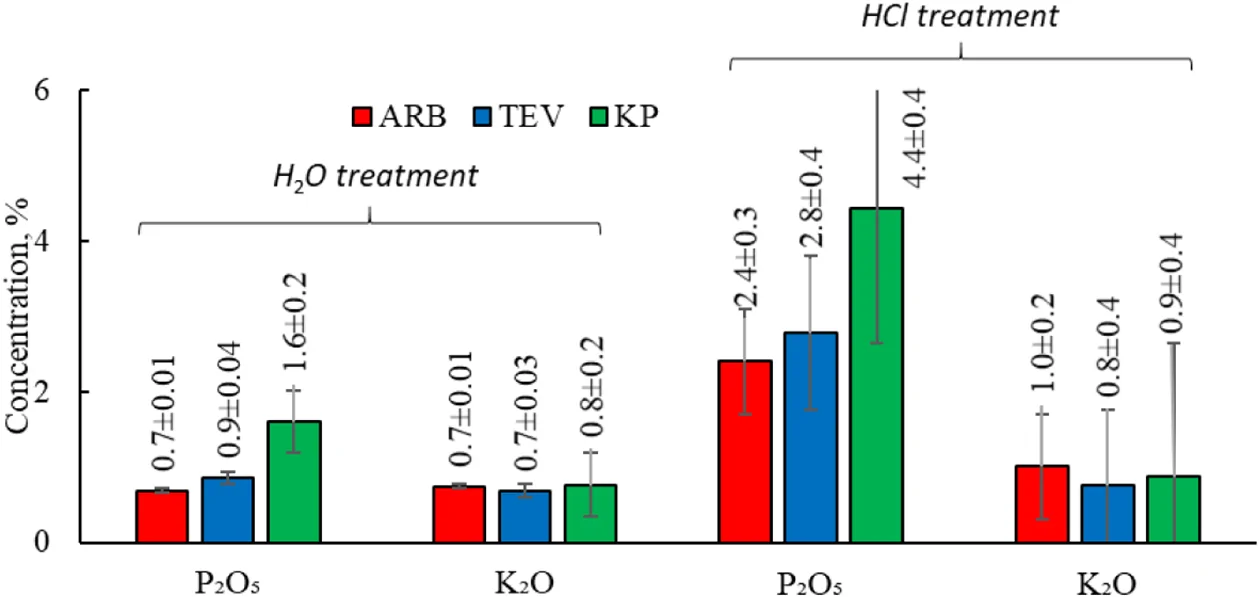
Phosphorus (P_2_O_5_) and potassium (K_2_O) content in the dried digestates after treatment with water or diluted hydrochloric acid. Values are presented as mean ± SD (*n* = 3).

Following water treatment, K_2_O contents were relatively similar among the digestates and ranged from 0.7% to 0.8% dry matter. HCl treatment resulted in only moderately higher values, with a maximum K_2_O content of approximately 1.0% in ARB. The relatively small differences between the two treatments suggest that a substantial proportion of the potassium quantified under these conditions was readily released by water treatment.

The P_2_O_5_ contents determined following HCl treatment in the present study ranged from 2.4% to 4.4%, whereas the corresponding K_2_O contents ranged from 0.8% to 1.0% of dry matter. Egene et al. reported total phosphorus and potassium contents equivalent to approximately 0.3–3.2% P_2_O_5_ and 0.1–1.1% K_2_O, respectively, in differently processed solid digestate fractions.^31^ The K_2_O contents determined in the present study were therefore within the reported range, whereas the P_2_O_5_ contents were near its upper limit or higher. However, direct comparison is limited because Egene et al. determined total element contents after mineralization, whereas the present results represent the fractions released following HCl treatment. Differences may also reflect variations in feedstock composition, digestion technology, operating conditions, and postdigestion processing. Similarly, Slepetiene et al. reported a broad range of total nutrient contents in different digestate phases, equivalent to approximately 0.5–4.2% P_2_O_5_ and 0.7–5.6% K_2_O, demonstrating the substantial compositional variability of digestate materials.^32^

In addition to the primary plant nutrients, calcium and magnesium were determined following treatment of the digestates with water or HCl. Following water treatment, the CaO concentrations were 0.09 ± 0.06, 0.07 ± 0.05, and 0.09 ± 0.03%, while the corresponding MgO concentrations were 0.12 ± 0.01, 0.62 ± 0.06, and 0.25 ± 0.03% for ARB, TEV, and KP, respectively. Following HCl treatment, the CaO concentrations increased to 1.7 ± 0.2, 3.5 ± 0.1, and 3.1 ± 0.1%, whereas the corresponding MgO concentrations reached 5.0 ± 0.2, 4.9 ± 0.2, and 6.0 ± 0.2%, respectively. The markedly higher concentrations determined after HCl treatment suggest that a substantial proportion of Ca and Mg was associated with poorly water-soluble forms, potentially including Ca- and Mg-containing phosphates, whose formation and solubility depend on the chemical conditions in the digestate.^33^

Because the study focused on the recovery of organic carbon, the organic matter and organic carbon contents of the dried digestates were also determined. After cooling and weighing the remaining ash, the organic matter contents were 88.9 ± 2.6% for ARB, 88.2 ± 1.2% for TEV, and 91.5 ± 0.9% for KP.

The organic carbon content of the dried digestate samples was determined using the sulfochromic oxidation method. The highest OC (59.5 ± 3.6%) was observed in the KP digestate, while the values for ARB and TEV were 52.9 ± 3.0% and 58.1 ± 2.9%, respectively.

To further characterize the digestates, TE and HM (Fe, Co, Mn, Cu, Zn, Cr, Ni, Pb, and Cd) were determined following treatment with water or HCl. Mn, Cu, Zn, Cr, and Pb were not detected following either treatment, whereas the measured Fe, Co, Ni, and Cd values are presented in Table 2.

**Table 2:** Amounts of Detected Trace Elements and Heavy Metals Released from the Digestates Following Treatment with Water or HCl^a^

	Released amount, mg/g dry digestate					
	ARB		TEV		KP	
TE and HM	H_2_O	HCl	H_2_O	HCl	H_2_O	HCl
Fe	3.65 × 10^–2^	2.26	1.45 × 10^–2^	3.91	8.14 × 10^–3^	1.05
Co	2.75 × 10^–2^	4.50 × 10^–3^	–	–	5.09 × 10^–4^	–
Ni	5.25 × 10^–2^	–	5.98 × 10^–2^	–	4.85 × 10^–2^	–
Cd	7.25 × 10^–3^	1.40 × 10^–2^	1.15 × 10^–2^	5.22 × 10^–2^	1.54 × 10^–2^	1.83 × 10^–2^

a Note: “–” indicates a value below the method detection limit.

Among the detected elements, Fe showed the highest released amounts, particularly following HCl treatment, reaching 2.26 mg/g in ARB, 3.91 mg/g in TEV, and 1.05 mg/g in KP. Ni was quantified only following water treatment, with the highest amount determined in TEV (5.98 × 10^–2^ mg/g), whereas it remained below the detection limit in the HCl-treated samples. Cd was detected following both treatments in all three digestates, with released amounts ranging from 7.25 × 10^–3^ to 5.22 × 10^–2^ mg/g. Co was quantified following water treatment in ARB and KP and following HCl treatment only in ARB.

Published total-content data demonstrate that the trace-element and heavy-metal composition of digestates varies substantially according to their origin and feedstock, with pronounced differences in Cd and Ni contents reported among digestates derived from sewage sludge, cattle manure, maize silage, and mixed agro-industrial wastes.^34^ Digestates produced mainly from source-separated organic and industrial food wastes have also been shown to contain Cd, Cu, and Zn at levels relevant to their quality classification, indicating that the use of food-waste-based feedstocks does not eliminate the need for contaminant assessment.^35^ Recent reviews likewise emphasize that the suitability of digestate for agricultural valorization should be evaluated on the basis of total heavy-metal contents and their comparison with the applicable regulatory limits.^36^

The values obtained in the present study represent the amounts released under the applied water and HCl treatment conditions rather than the total elemental contents of the digestates. Therefore, they cannot be directly compared with literature values obtained following complete mineralization or with regulatory limits based on the total content. Accordingly, complete mineralization and determination of total element contents would be required before the environmental safety of these digestates or their suitability for soil application could be assessed.

### 3.3 Instrumental Characterization of the Digestates

Simultaneous thermal analysis (STA), combining thermogravimetric (TG) and differential scanning calorimetric (DSC) measurements, showed broadly similar thermal behavior for all three digestates (Figure 5). The first mass-loss stage extended to approximately 130–150 °C and was attributed mainly to the removal of physically adsorbed and weakly bound moisture. The associated mass losses were 8.7% for ARB, 7.0% for TEV, and 7.0% for KP. This stage was accompanied by endothermic effects, with DSC minima observed at 91.2 °C for ARB, 131.0 °C for TEV, and 99.2 °C for KP. The major thermal degradation region extended approximately from 200 to 600 °C and consisted of several overlapping thermal events accompanied by the steepest decrease in the TG curves. For ARB (Figure 5a), the DSC maxima were observed at 324.2 and 393.1 °C, followed by additional thermal effects at 484.5 and 510.6 °C. For TEV digestate (Figure 5b), the corresponding peaks were detected at 324.6 and 410.0 °C, with further thermal effects at 468.7 and 505.2 °C. For KP sample (Figure 5c), the first two major peaks were shifted toward higher temperatures and were recorded at 333.5 and 430.1 °C, followed by additional peaks at 473.6 and 499.6 °C. The main mass-loss step shown in Figure 5 amounted to 62.8% for ARB, 64.3% for TEV, and 71.6% for KP, indicating that KP exhibited the largest thermally decomposable fraction in this temperature range.

**Figure 5:**
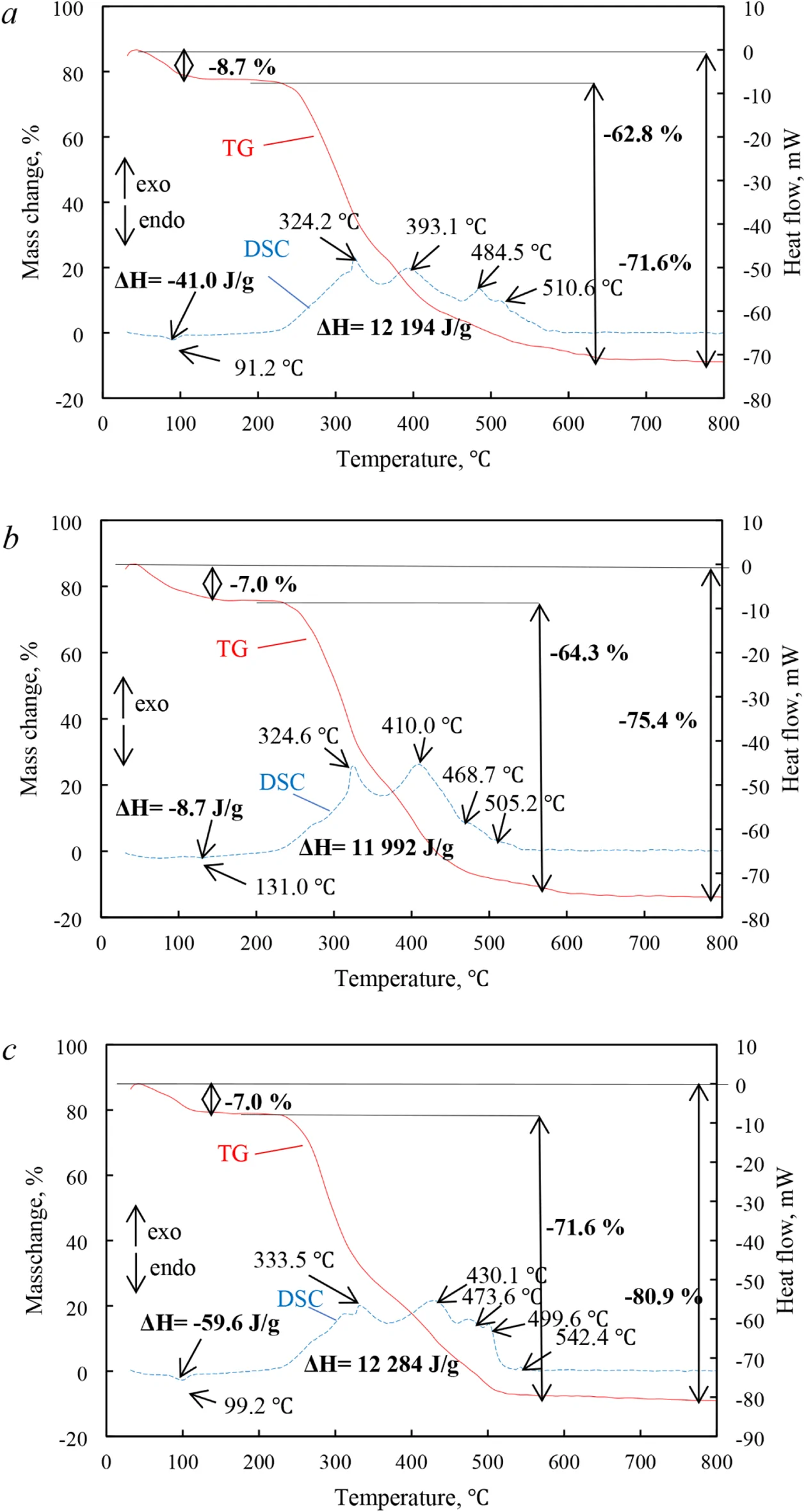
Simultaneous thermal analysis of dried digestates: a: ARB, b: TEV, and c: KP.

The TG curves approached nearly constant values at approximately 550–600 °C. The total mass loss measured by 800° reached 71.6% for ARB, 75.4% for TEV, and 80.9% for KP, corresponding to residual solid fractions of 28.4, 24.6, and 19.1%, respectively. Thus, KP exhibited the largest thermally decomposable fraction and the lowest residual solid fraction under the applied experimental conditions. However, the higher mass loss should not be attributed exclusively to organic matter, because transformations of inorganic constituents may also contribute within this temperature range.

The observed TG–DSC profiles are consistent with the multistage thermal decomposition reported for solid digestates. Karaeva et al. identified drying, volatile release, and decomposition of carbonaceous residues, with the greatest mass loss occurring at approximately 180–425 °C. This agrees with the major thermal effects observed in the present study at 324–430 °C.^37^ Swiechowski et al. reported a higher-temperature decomposition region of approximately 350–550 °C, corresponding to the additional effects observed here at 469–511 °C. These variations may be related to differences in feedstock composition, mineral content, and thermal-analysis conditions.^38^ Bartocci et al. also demonstrated the complex, multistep pyrolysis behavior of lignin-rich solid digestate. Therefore, the overlapping DSC effects observed in the present study cannot be assigned unambiguously to individual lignocellulosic components.^39^

XRD analysis results of the digestate samples are shown in Figure 6. The ARB and TEV digestates (Figure 6a,b) were dominated by broad diffuse halos, although several weak and narrow reflections were also visible, indicating that these samples are predominantly in an amorphous phase with a relatively limited crystalline contribution.

**Figure 6:**
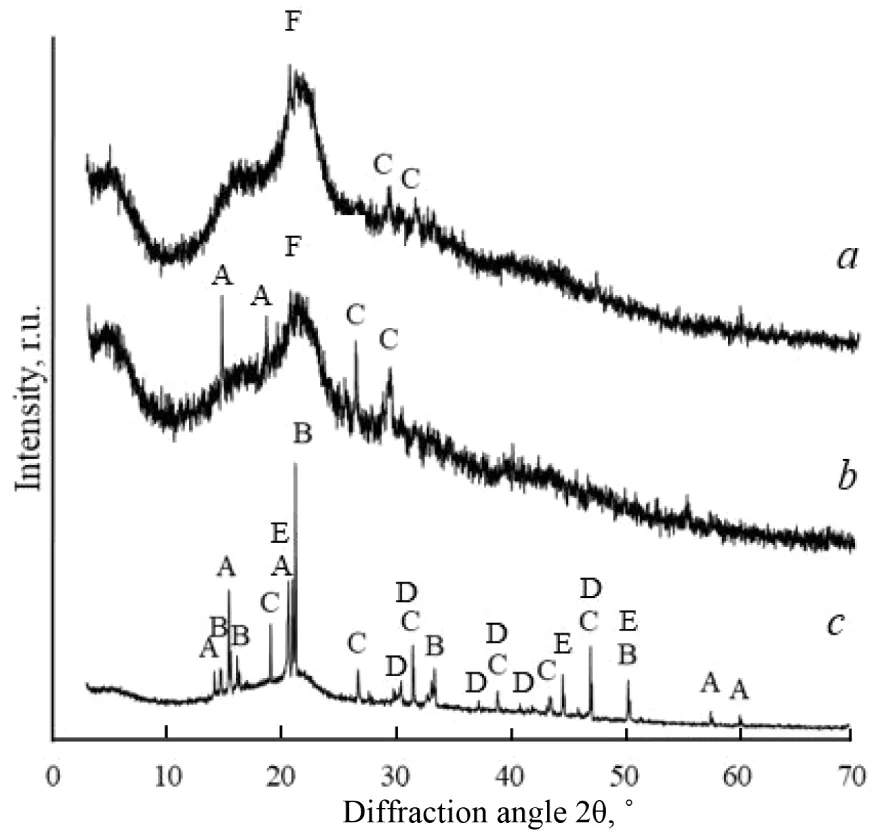
X-ray diffraction patterns of digestates: a: ARB, b: TEV, and c: KP; tentative database matches indicated candidate phases: A: phosphate-related mineral phases (e.g., MgNH_4_PO_4_·6H_2_O), B: magnesium-containing salts (e.g., MgSO_4_ hydrates), C: potassium sulfate-type salts (e.g., K_2_SO_4_), D: calcite (CaCO_3_), E: quartz (SiO_2_), and F: broad halo associated with poorly ordered organic material.

In contrast, the KP digestate (Figure 6c) shows several distinct diffraction peaks, indicating the presence of crystalline phases. Tentative phase identification was performed using the RSDA Search-Match database by comparing the detected reflections to reference patterns and considering the elemental composition determined by chemical analysis. The database matches suggested the possible presence of carbonate-, phosphate-, and sulfate-containing salts and other Mg-, K-, and ammonium-containing crystalline phases.

Duong et al.^40^ similarly identified amorphous and crystalline cellulose together with inorganic crystalline phases, including quartz, CaCO_3_, KCl, CaS, and Mg_3_(PO_4_)_2_, in solid biogas digestate. The occurrence of reflections in comparable 2θ regions is consistent with the possible presence of carbonate- and phosphate-containing phases in the KP digestate, although direct phase equivalence cannot be confirmed because of differences in feedstock composition and overlapping diffraction peaks. Yazıcı et al.^41^ confirmed struvite in precipitates produced from liquid digestate under induced crystallization conditions. Therefore, their results support the possibility of struvite formation from digestate but do not directly confirm its presence in the untreated KP sample.

The differences in diffraction patterns indicate that the KP digestate exhibited a more pronounced crystalline contribution than did the ARB and TEV samples. This observation may be associated with differences in the mineral composition of the samples and is consistent with the higher concentrations of selected mineral nutrients determined in the KP. The coexistence of poorly ordered organic structures and distinct crystalline mineral phases has also been reported for solid biogas digestates.^40^ Differences among the samples may reflect variations in feedstock composition, mineral speciation, and digestate processing conditions.

FT-IR spectroscopy has been used to characterize organic matter and identify functional groups in digestate materials. As shown in Figure 7, the spectra of all three digestates were generally similar, indicating the presence of comparable functional groups. The broad absorption region between approximately 3600 and 3200 cm^–1^ was assigned to overlapping O–H and N–H stretching vibrations associated with hydroxyl groups, adsorbed or bound water, and nitrogen-containing compounds. Absorption bands in the approximately 1700–1600 cm^–1^ region may be related to overlapping C=O and aromatic C=C stretching vibrations and H–O–H bending vibrations. Bands at approximately 1500–1400 cm^–1^ were tentatively associated with C–H and/or NH_4_^+^ vibrations.^24,42^

**Figure 7:**
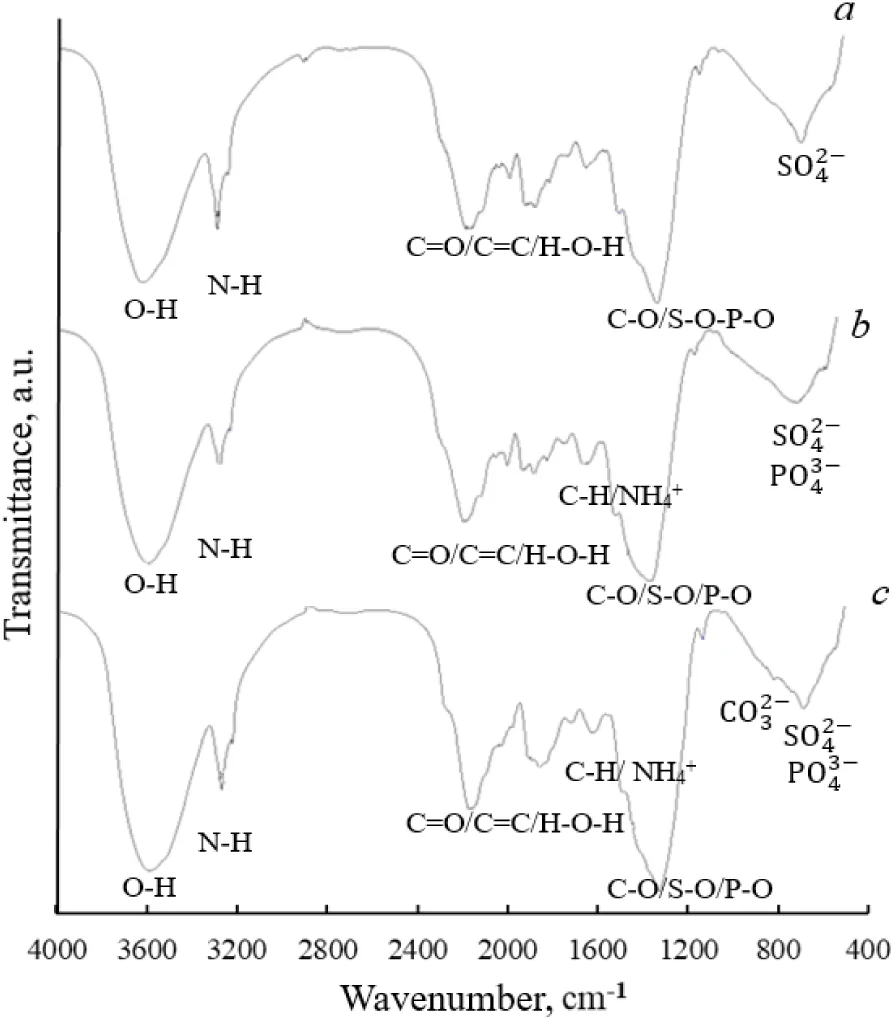
FT-IR spectra of digestates: a: ARB, b: TEV, and c: KP.

The strong absorption region between approximately 1200 and 1000 cm^–1^ was attributed to overlapping C–O, S–O, and P–O stretching vibrations, suggesting contributions from both organic matter and sulfate- or phosphate-containing components. Bands in the lower wavenumber region, approximately 700–500 cm^–1^, were tentatively assigned to SO_4_^2–^ and PO_4_^3–^ vibrations. Similar sulfate- and phosphate-containing compounds were reported in acidified food-waste digestate products, although these materials were obtained under different processing conditions.^43^

An additional band associated with CO_3_^2–^ was observed in the KP spectrum. This observation supports the possible presence of carbonate-containing phases indicated by XRD analysis and is consistent with carbonate-related bands previously reported for stabilized digestate.^42^ Overall, the FT-IR results support the coexistence of organic functional groups and sulfate-, phosphate-, and possibly carbonate-containing inorganic components. However, because several absorption bands overlap, the exact mineral salts cannot be identified by FT-IR alone.

Overall, the instrumental analyses revealed both common features and clear structural differences among the digestates. All samples showed similar multistage thermal behavior and comparable organic functional groups, whereas KP exhibited the largest thermally decomposable fraction, the lowest residual solid fraction, and a more pronounced crystalline contribution. The observed differences among the digestates may partly be related to their contrasting feedstock compositions. ARB was produced from manure, straw, slaughterhouse residues, and green biomass, whereas TEV originated from food products, oils, slaughterhouse residues, and restaurant waste; these heterogeneous inputs may have contributed to the more pronounced fibrous structures observed in both materials. In contrast, KP, produced mainly from grain residues and food-processing waste, exhibited a more compact morphology and a more pronounced crystalline contribution. However, because the feedstocks were complex and the operating conditions of the biogas plants were not controlled in this study, individual structural or chemical features cannot be attributed to any single feedstock component. The combined XRD and FT-IR results indicated the coexistence of poorly ordered organic matter with phosphate-, sulfate-, and possibly carbonate-containing inorganic components, with their relative contributions varying among the samples.

### 3.4 Effect of Extraction Conditions on Organic Carbon Recovery from Digestate

After characterizing the physicochemical properties, the digestates were used for the preparation of aqueous and alkaline extracts to evaluate organic carbon extraction efficiency. On the basis of our research data and findings reported by other authors, digestates contain high concentrations of organic carbon. To maximize organic carbon extraction from the three digestates, experiments were conducted under different conditions, including raw material preparation, temperature, extraction time, extraction method, and extractant type.

Two extractants were used: water and 0.5 M potassium hydroxide. Extraction durations of 3, 6, and 9 days were investigated. The results (Figure 8) show marked differences in TOC concentrations between the aqueous and alkaline extracts. The use of potassium hydroxide resulted in TOC concentrations approximately 8–29 times those measured in aqueous extracts. After 3 days of extraction in 0.5 M KOH, TOC concentrations reached: 1802–1830 mg/L for ARB, 1449–1894 mg/L for TEV, and 1684–1913 mg/L for KP. In contrast, the corresponding aqueous extracts contained substantially lower concentrations: ARB, 99–116 mg/L; TEV, 67–79 mg/L; and KP, 175–254 mg/L. The higher TOC concentrations in KOH extracts may be attributed to enhanced solubilization of organic matter, including humic-like substances, under alkaline conditions. In a recent digestate valorization study, ammonia used as an alkaline extraction medium significantly increased the solubility of organic matter in the digestate residue.^9^ Alkali-hydrothermal treatment has likewise been shown to promote the solubilization and release of dissolved organic matter from anaerobic digestion sludge.^44^

**Figure 8:**
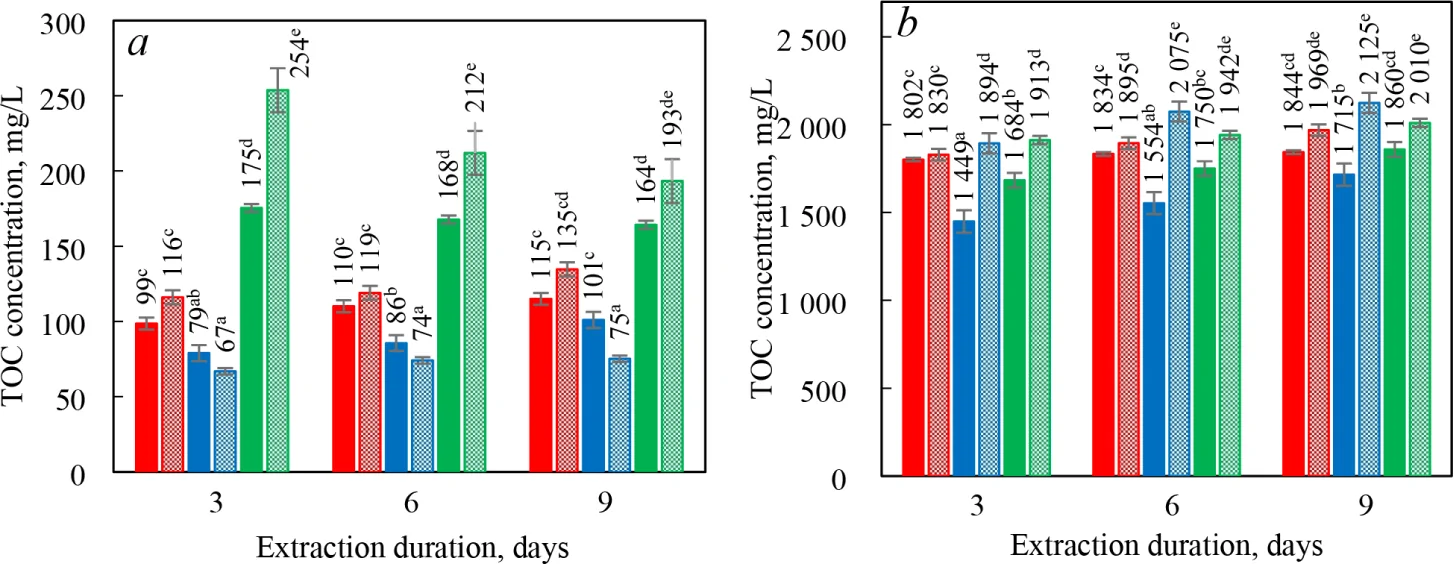
Total organic carbon (TOC) concentration in digestate extracts obtained using a: H_2_O and b: KOH after 3, 6, and 9 days of extraction. Values are presented as mean ± SD of three independently prepared extracts (*n* = 3). Different lowercase letters indicate statistically significant differences among means within the same panel according to Tukey’s HSD test (*p* < 0.05).

The effect of crushing on the TOC concentration was condition- and digestate-dependent. As shown in Figure 8a, KP digestate (both WD and CD) exhibited higher TOC concentrations in aqueous extracts than those of ARB and TEV. For ARB, crushing did not significantly affect the TOC concentrations of the aqueous extracts. For TEV, the CD values were lower than the WD values with statistically significant differences observed after 6 and 9 days. In contrast, KP CD exhibited significantly higher TOC concentrations than KP WD after 3 and 6 days, whereas no significant difference was observed after 9 days.

In the KOH extracts (Figure 8b), TOC concentrations generally increased with extraction time. A statistically significant increase between 3 and 9 days was observed for ARB CD, TEV WD and CD, and KP WD and CD, whereas ARB WD did not change significantly. Numerically, the effect of crushing was most pronounced for TEV, for which the CD extracts exhibited significantly higher TOC concentrations than the WD extracts at all extraction times. For KP, the CD extracts also exhibited significantly higher TOC concentrations than the WD extracts at all three extraction times, although the differences were smaller than those observed for TEV. For ARB, a significant difference between the WD and CD extracts was observed only after 6 days. Thus, the extractant type produced the largest numerical difference in TOC concentration, while the effects of crushing and extraction time varied among the digestates. The limited and condition-dependent effect of crushing suggests that, within the investigated particle size ranges (<2 and 3–5 mm), chemical solubilization and digestate matrix composition were generally more influential than mass-transfer limitations. The relatively high liquid-to-solid ratio and long extraction times may also have reduced the additional benefit of particle size reduction.

Overall, the results indicate that the effects of sample preparation and extraction time depend on the digestate type. These effects were more evident in KOH extracts, particularly for the TEV. Within this static extraction series, the highest TOC concentrations in KOH extracts were generally obtained from crushed digestates after 9 days. A comparable effect of the alkaline extractant was reported for sewage sludge- and digestate-derived biochars, where NaOH extraction produced higher DOC concentrations than deionized or hot-water extraction; however, direct quantitative comparison is limited because the materials used in that study had undergone pyrolysis.^45^

Further experiments were performed using magnetic stirring at different temperatures and extraction times. The samples were stirred for 3, 6, and 9 h at room temperature (23 °C) and at elevated temperatures of 50, 70, and 90 °C. The obtained results are presented in Figure 9a–h.

**Figure 9:**
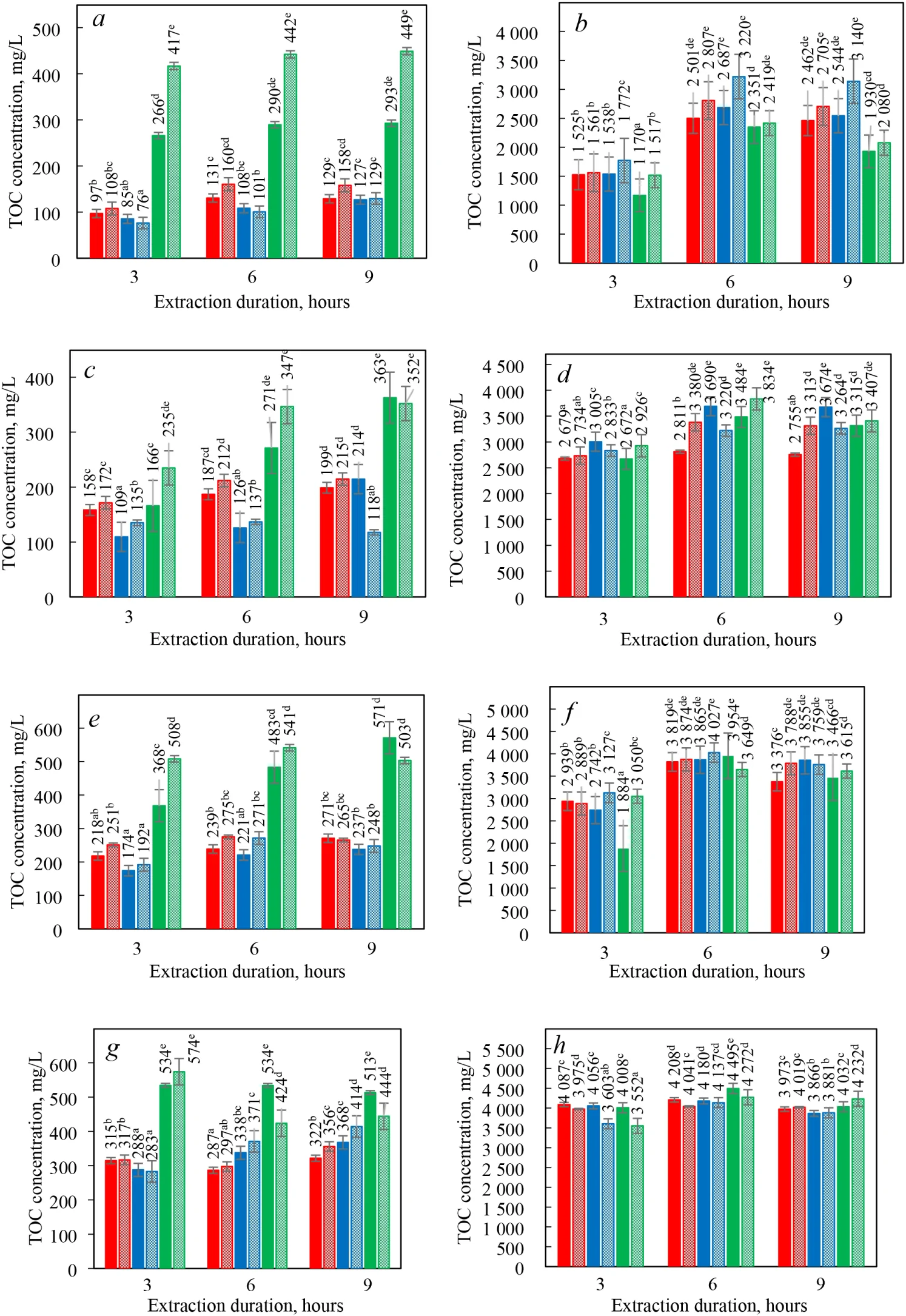
Total organic carbon (TOC) concentrations in digestate extracts obtained by magnetic stirring: a, c, e, g: H_2_O; b, d, f, h: KOH, and at temperatures: a, b: 23 °C; c, d: 50 °C; e, f: 70 °C, and g, h: 90 °C. Values are presented as mean ± SD of three independently prepared extracts (*n* = 3). Different lowercase letters indicate statistically significant differences among means within the same panel according to Tukey’s HSD test (*p* < 0.05).

When comparing extracts produced at different temperatures, it was observed that increasing the temperature generally resulted in higher TOC concentrations in both aqueous and potassium hydroxide extracts, although the effect depended on the type of digestate and extraction conditions.

At the highest temperature (90 °C), the highest TOC concentrations in the aqueous extracts were observed for the KP digestate samples (Figure 9g). In the KOH extracts obtained at 90 °C (Figure 9h), the highest overall TOC concentration was recorded for KP WD after 6 h of stirring (4495 mg/L), while KP CD reached 4272 mg/L under the same conditions.

In all KOH extraction series (Figure 9b,d,f,h), the highest numerical TOC concentrations for each digestate variant were obtained after 6 h of stirring. However, the differences between 6 and 9 h were not always statistically significant. For example, in the TEV CD sample at 70 °C (Figure 9f), the TOC concentration reached 4027 mg/L after 6 h of stirring, whereas a concentration of 3759 mg/L was obtained after 9 h. These values did not differ significantly, indicating that prolonging the extraction from 6 to 9 h did not provide a further significant increase in TOC concentration.

In aqueous extracts, TOC concentrations often increased with stirring time, particularly at 23 and 50 °C (Figure 9a,c), although the response was less consistent at 70 and 90 °C (Figure 9e,g). For example, in the KP CD digestate extracted with water at 70 °C (Figure 9e), the TOC concentration decreased numerically from 541 mg/L after 6 h to 503 mg/L after 9 h, although the difference was not statistically significant. In the KP CD sample extracted at 90 °C (Figure 9g), the TOC concentration decreased significantly from 574 mg/L after 3 h to 424 and 444 mg/L after 6 and 9 h, respectively. No significant difference was observed between the latter two values. For KP WD at 90 °C (Figure 9g), the TOC concentrations of 534, 534, and 513 mg/L obtained after 3, 6, and 9 h, respectively, did not differ significantly.

Overall, the results demonstrate that both the temperature and stirring time are important factors influencing the TOC concentrations obtained in the extracts. Elevated temperatures generally enhanced the solubilization of organic carbon, particularly in alkaline media, while the effect of extraction time depended on the digestate type and extraction conditions. For the KOH extracts, 6 h of stirring consistently produced the highest numerical TOC concentrations, while prolonging the process to 9 h generally provided no additional improvement.

These observations are consistent with studies applying a combined alkaline and hydrothermal treatment to anaerobic digestion residues. Du et al. reported that alkali-hydrothermal treatment promoted the solubilization and release of dissolved organic matter from anaerobic digestion sludge.^44^ Similarly, Han et al. demonstrated that temperature, treatment time, and KOH dosage influenced the production of humic acids from corn straw digestate under alkaline hydrothermal conditions.^11^ Although the treatment conditions applied in that study were more intensive than those used in the present work, the findings support the combined influence of alkaline conditions, temperature, and treatment duration on the solubilization and transformation of digestate organic matter. In the present study, the combination of 0.5 M KOH, elevated temperature, and magnetic stirring resulted in TOC concentrations of up to approximately 4500 mg/L.

Additional experiments were performed using an ultrasonic bath. The same extraction durations as in the magnetic stirring experiments were maintained, but temperatures of 30, 50, and 60 °C were chosen because of the operating range of the ultrasonic bath. The obtained results are presented in Figure 10.

**Figure 10:**
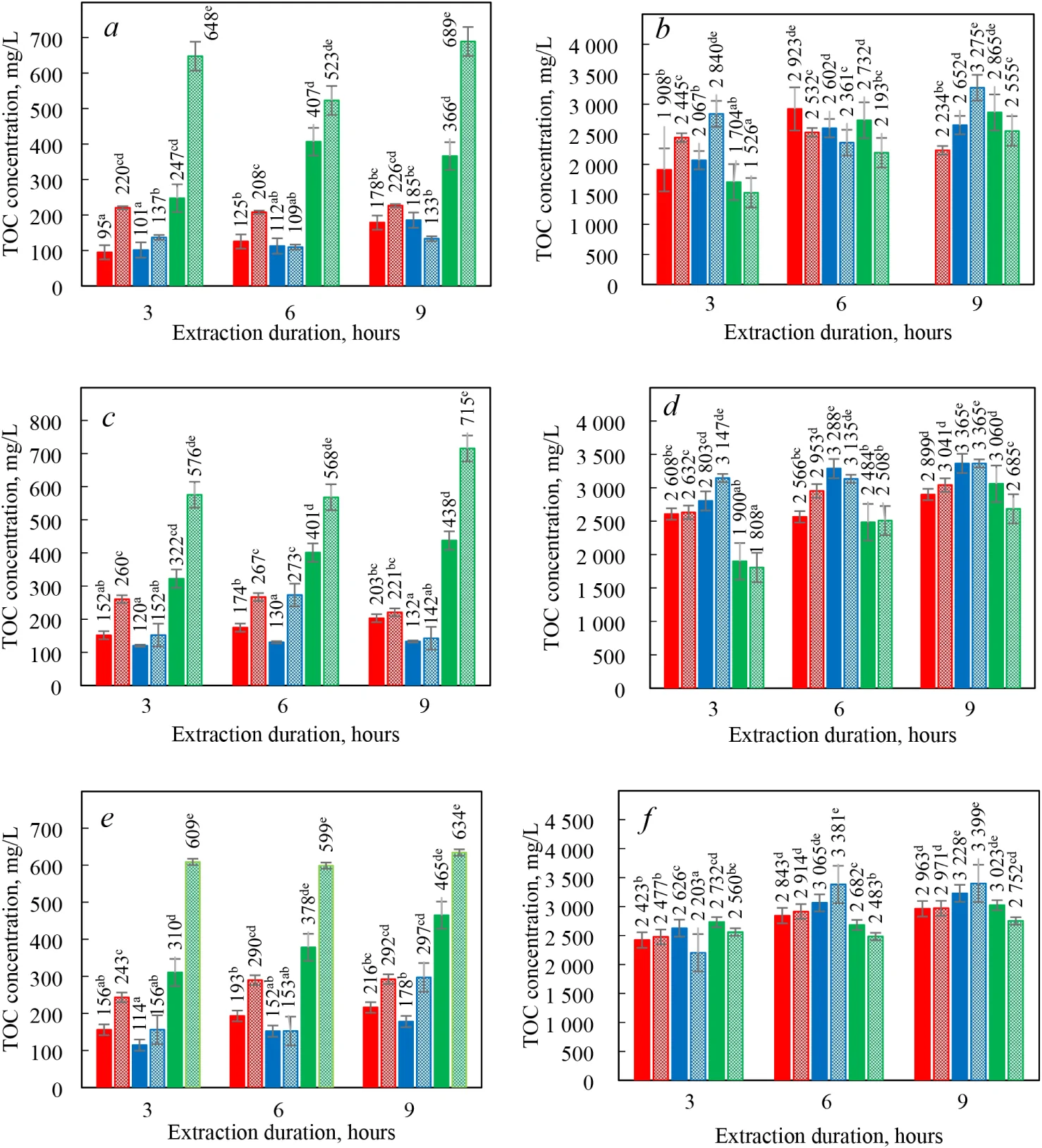
Total organic carbon (TOC) concentrations in digestate extracts obtained using an ultrasonic bath: a, c, e: H_2_O; b, d, f: KOH, and temperature: a, b: 30 °C; c, d: 50 °C; e, f: 60 °C. Values are presented as mean ± SD of three independently prepared extracts (*n* = 3). Different lowercase letters indicate statistically significant differences among means within the same panel according to Tukey’s HSD test (*p* < 0.05).

In aqueous extracts (Figure 10a,c,e), the highest TOC concentration was observed for the KP CD sample at 50 °C, reaching 715 mg/L after 9 h of extraction (Figure 10c). This value was numerically higher than the maximum obtained for the same sample using magnetic stirring at 90 °C, which reached 574 mg/L after 3 h (Figure 9g). However, the conditions used in these two-extraction series were not identical, and the values were not compared statistically.

The effect of extraction time in aqueous media depended on the digestate type and temperature. At 30 °C (Figure 10a), the TOC concentrations of ARB WD and TEV WD increased significantly between 3 and 9 h. In contrast, the highest numerical concentration for KP WD was obtained after 6 h, whereas KP CD reached its highest numerical concentration after 9 h. For KP CD, however, the values obtained after 3, 6, and 9 h did not differ significantly. At 50 °C (Figure 10c), TEV CD reached its highest TOC concentration after 6 h, followed by a significant decrease after 9 h. At 60 °C (Figure 10e), the TOC concentrations of TEV WD and TEV CD were significantly higher after 9 h than after 3 h. Although KP CD exhibited the highest numerical TOC concentration after 9 h at all three temperatures, the effect of extraction time on this sample was not statistically significant within any of the aqueous extraction panels.

Figure 10b,d,f shows that substantially higher TOC concentrations were obtained in the potassium hydroxide extracts than in the corresponding aqueous extracts. Longer extraction times generally resulted in higher TOC concentrations in KOH media, particularly at 50 and 60 °C (Figure 10d,f), although the response remained dependent on the digestate type. For example, the TEV CD sample extracted at 60 °C for 9 h reached a TOC concentration of 3399 mg/L (Figure 10f). This value did not differ significantly from the 3381 mg/L obtained after 6 h, indicating that prolonging the extraction from 6 to 9 h did not significantly improve TOC recovery for this sample.

At 9 h, the highest numerical TOC concentration in the KOH extracts increased slightly from 3275 mg/L at 30 °C (Figure 10b) to 3365 mg/L at 50 °C (Figure 10d) and 3399 mg/L at 60 °C (Figure 10f); all three values were obtained for TEV CD. However, because the statistical analysis was performed separately within each panel, the differences between temperatures cannot be considered statistically significant and represent only a numerical tendency.

Similar effects of ultrasonic treatment on the release of dissolved organic matter have been reported in previous studies. Ultrasonic cavitation may disrupt the solid organic matrix and facilitate the transfer of soluble organic compounds into the liquid phase. Zhang et al.^46^ showed that ultrasonic treatment induced the leaching of dissolved organic matter from sludge into the liquid phase and altered its molecular composition. Nieweś and Marecka^47^ demonstrated that the efficiency of ultrasound-assisted alkaline extraction of humic-like substances from compost and spent coffee grounds depended on extraction time, ultrasound intensity, NaOH concentration, and temperature. Shiju et al. most directly demonstrated the feasibility of ultrasound-assisted recovery of humic and fulvic substances from municipal digestate and evaluated the influence of ultrasound amplitude and extraction time.^48^ Although the raw materials, extraction conditions, and target organic fractions differed among these studies, their results support the use of ultrasound to intensify the transfer of organic matter into the liquid phase.

Compared with conventional magnetic stirring, the effect of ultrasonic treatment depended strongly on the extractant. In aqueous media, the maximum TOC concentration obtained using the ultrasonic bath was 715 mg/L for KP CD at 50 °C (Figure 10c), compared with 574 mg/L obtained using magnetic stirring at 90 °C (Figure 9g). In contrast, the maximum TOC concentration in KOH extracts was lower with ultrasonic treatment—3399 mg/L for TEV CD at 60 °C (Figure 10f)—than with magnetic stirring, which produced 4495 mg/L for KP WD at 90 °C (Figure 9h). Therefore, ultrasonic treatment enhanced organic carbon transfer under selected aqueous extraction conditions but cannot be considered generally more effective than magnetic stirring for all digestates and extractants. The different effects of ultrasonication and magnetic stirring may result from the dominant extraction mechanism in each medium. In water, ultrasonic cavitation can enhance mass transfer and disrupt the digestate structure, whereas in 0.5 M KOH, the strong chemical solubilization of organic matter, combined with heating and continuous mixing, may reduce the additional benefit of ultrasound.

The effect of temperature on the concentration of organic carbon in digestate extracts was also investigated. The extracts were prepared using the same solvents and extraction durations as in the previous experiments, but under constant-temperature conditions without additional mixing. The temperatures corresponded to those used in the magnetic stirring experiments (50, 70, and 90 °C). The obtained results are presented in Figure 11a–f.

**Figure 11:**
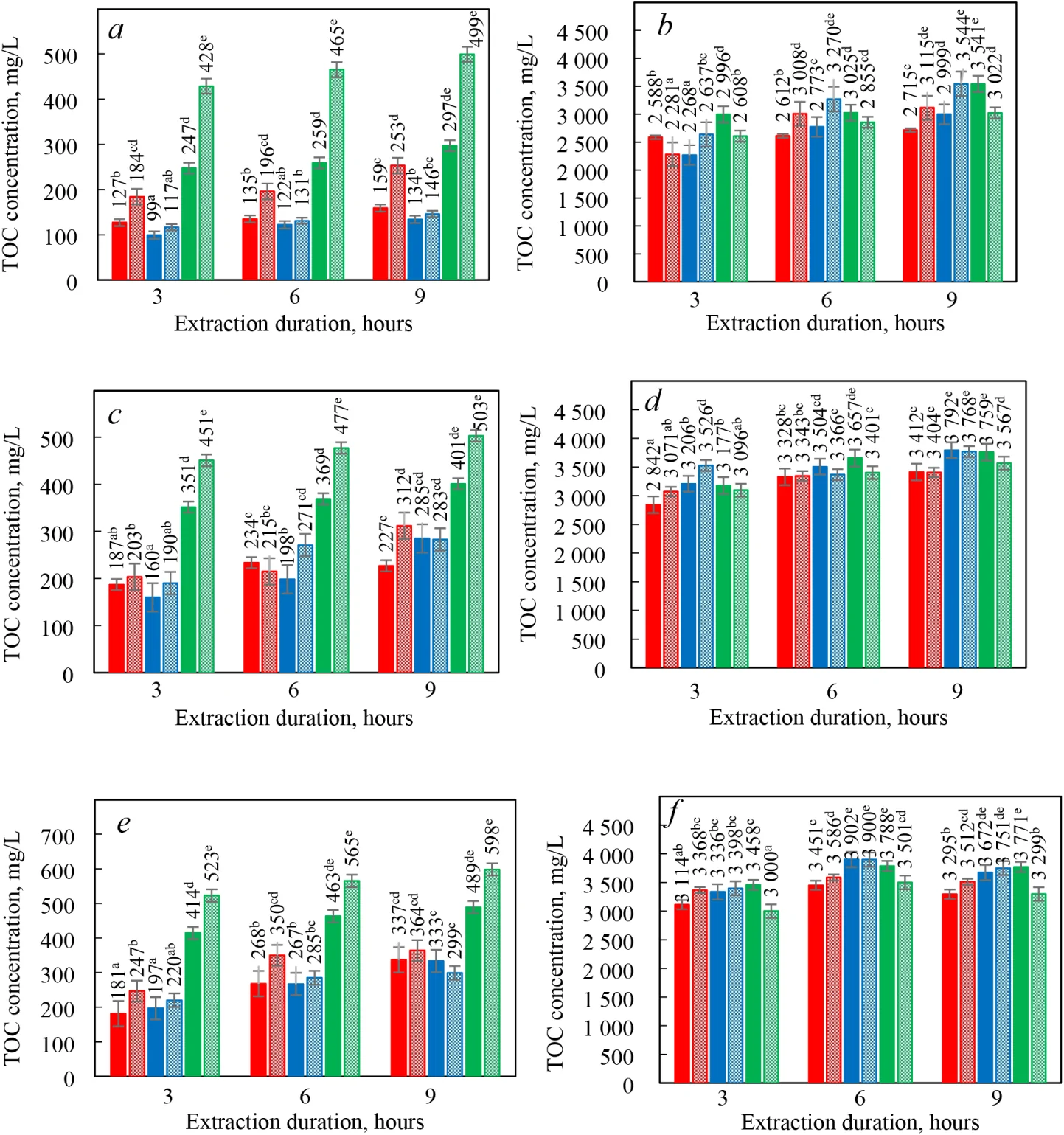
Total organic carbon (TOC) concentrations in digestate extracts obtained under temperature-controlled conditions without additional mixing: a, c, e: H_2_O; b, d, f: KOH, and temperatures: a, b: 50 °C; c, d: 70 °C; e, f: 90 °C. Values are presented as mean ± SD of three independently prepared extracts (*n* = 3). Different lowercase letters indicate statistically significant differences among means within the same panel according to Tukey’s HSD test (*p* < 0.05).

TOC concentrations generally increased numerically with an increase in temperature, although the response depended on the digestate type, extractant, and extraction duration. Because the statistical analysis was performed separately within each panel, differences among temperatures were not evaluated statistically.

At 50 and 70 °C, the highest numerical TOC concentrations for all digestate variants in the KOH extracts were observed after 9 h of extraction (Figure 11b,d). For example, at 50 °C (Figure 11b), the highest concentrations for the respective digestates were recorded for ARB CD (3115 mg/L), TEV CD (3544 mg/L), and KP WD (3541 mg/L). At 70 °C (Figure 11d), the highest values for the respective digestates were observed for ARB WD (3412 mg/L), TEV WD (3792 mg/L), and KP WD (3759 mg/L).

However, at 90 °C, all KOH extract variants reached their highest numerical TOC concentrations after 6 h (Figure 11f). Prolonging the extraction time to 9 h resulted in lower numerical TOC concentrations. Therefore, under these conditions, extending the extraction from 6 to 9 h did not provide any additional improvement in organic carbon extraction. However, the obtained results alone do not provide sufficient evidence to attribute this decrease to the degradation or transformation of dissolved organic compounds.

In the aqueous extracts, TOC concentrations generally increased with increasing extraction time, particularly at 50 and 90 °C (Figure 11a,e), whereas the response at 70 °C was slightly less consistent (Figure 11c). For example, the TOC concentration in ARB WD at 70 °C increased from 187 mg/L after 3 h to 234 mg/L after 6 h and then decreased slightly to 227 mg/L after 9 h (Figure 11c). In KP CD aqueous extract, the TOC concentration increased from 428 to 499 mg/L at 50 °C (Figure 11a), from 451 to 503 mg/L at 70 °C (Figure 11c), and from 523 to 598 mg/L at 90 °C (Figure 11e) when the extraction time was extended from 3 to 9 h.

The observed effects of alkaline conditions and temperature are consistent with those of previous studies on anaerobic digestion residues. Du et al. reported that alkali-hydrothermal treatment promoted the solubilization and release of dissolved organic matter from anaerobic digestion sludge.^44^ Han et al. Similarly, they demonstrated that temperature, treatment time, and KOH dosage influenced humic acid production from corn straw digestate under alkaline hydrothermal conditions.^11^ Although the conditions applied in those studies were more intensive than those used in the present work, their results support the combined influence of alkaline conditions, temperature, and treatment duration on the transfer and transformation of digestate organic matter.

All digestate extracts were analyzed to determine their pH values. The aqueous extracts were close to neutral with pH values ranging from 6.3 to 7.5. As expected, potassium hydroxide extracts were strongly alkaline with pH values between 12.8 and 13.4.

The electrical conductivity (EC) of all digestate extracts was determined to characterize the overall ionic load of the resulting solutions and to assess the influence of the extraction medium and procedure. The results indicated that variation among the extraction procedures within each extractant group was limited, whereas the type of extractant had a clear influence. The EC values of the aqueous digestate extracts ranged from 9.8 ± 0.1 to 11.3 ± 0.1 mS/cm, indicating relatively similar ionic loads in the extracts obtained using water. These values are comparable to the EC of 9.27 mS/cm previously reported for an aqueous digestate suspension prepared at a solid-to-liquid ratio of 1:20. A higher EC value of 16.46 mS/cm was reported for the aqueous phase obtained after hydrothermal treatment of digestate and was associated with an increased concentration of dissolved ions and salts.^49^ Electrical conductivity values of 8.34 and 10.99 mS/cm have also been reported for digestates of different origins, while the generally high salt content of digestates has been identified as a major factor contributing to their conductivity.^50^ Thus, the EC values determined for the aqueous extracts in the present study are within the range expected for digestate-derived aqueous systems.

In contrast, the alkaline extracts prepared with 0.5 M KOH exhibited substantially higher EC values, ranging from 68.9 ± 0.2 to 70.1 ± 0.2 mS/cm. Published physicochemical data for aqueous KOH solutions demonstrate that their electrical conductivity is strongly dependent on KOH concentration and temperature, confirming the substantial contribution of the alkaline medium to the conductivity of the resulting extracts.^51^ The limited variation in EC within each extractant group suggests that the different extraction procedures had only a minor influence on the measured conductivity, whereas the extraction medium was the dominant factor. The markedly higher EC observed in the alkaline extracts can therefore be attributed primarily to the high ionic background introduced by 0.5 M KOH, particularly K^+^ and residual OH^–^ ions, together with ionic species released from or formed through interactions with the digestate matrix during alkaline extraction. However, because EC depends not only on ion concentration but also on ion identity, mobility, and measurement temperature, it should be interpreted as an indicator of the overall ionic load rather than as a direct measure of the total ion concentration.

The high pH and EC values of the KOH extracts should be considered when evaluating their potential agronomic applications. High salt concentrations may limit the direct use of digestate-derived materials,^50^ and previous plant experiments with a different digestate-derived liquid fraction showed that high EC and alkalinity could adversely affect plant growth.^49^ Therefore, the KOH extracts obtained in the present study cannot be considered ready-to-use liquid fertilizers without further evaluation of dilution or neutralization requirements, nutrient availability, and plant response.

Overall, the TOC concentrations obtained in the extracts were influenced by the temperature, extraction time, extractant type, and extraction method. Alkaline conditions and elevated temperatures generally resulted in higher TOC concentrations, although the optimal extraction duration depended on the digestate type and the temperature.

### 3.5 Carbon Recovery Efficiency under Selected Extraction Conditions

Based on the measured total organic carbon (TOC) concentrations, the mass of organic carbon recovered per gram of dry digestate and the corresponding carbon recovery efficiency relative to the initial organic carbon (OC) content were calculated. For comparison, the conditions yielding the highest TOC concentrations within each experimental series were selected. These conditions covered the extractant type (H_2_O or 0.5 M KOH), extraction mode (static extraction, temperature-controlled extraction without additional mixing, magnetic stirring, and ultrasonication), temperature (23–90 °C), and extraction duration (3, 6, and 9 days or 3, 6, and 9 h). The calculated carbon recovery values are presented in Table 3. Carbon recovery efficiency was calculated using the initial OC contents of 58.1% for TEV and 59.5% for KP, corresponding to 0.581 and 0.595 g C/g dry digestate, respectively.

**Table 3:** Organic Carbon Recovery under Selected Digestate Extraction Conditions

Digestate	Extraction conditions	TOC, mg/L	*m*_C,rec_, mg C/g dry digestate	η_C_, %
TEV WD	KOH, 9 h, 70 °C	3792	379	65.3
	KOH, 6 h, 90 °C	3902	390	67.2
	KOH, 9 h, 50 °C, ultrasonic bath	3365	337	57.9
TEV CD	KOH, 9 days, 23 °C	2125	213	36.6
	KOH, 9 h, 50 °C	3544	354	61.0
	KOH, 6 h, 23 °C, stirring	3220	322	55.4
	KOH, 6 h, 70 °C, stirring	4027	403	69.3
	KOH, 9 h, 30 °C, ultrasonic bath	3275	328	56.4
	KOH, 9 h, 50 °C, ultrasonic bath	3365	337	57.9
	KOH, 9 h, 60 °C, ultrasonic bath	3399	340	58.5
KP WD	H_2_O, 9 h, 50 °C, stirring	363	36	6.1
	H_2_O, 9 h, 70 °C, stirring	571	57	9.6
	KOH, 6 h, 90 °C, stirring	4495	450	75.6
KP CD	H_2_O, 3 days, 23 °C	254	25	4.3
	H_2_O, 9 h, 50 °C	499	50	8.4
	H_2_O, 9 h, 70 °C	503	50	8.5
	H_2_O, 9 h, 90 °C	598	60	10.1
	H_2_O, 9 h, 23 °C, stirring	449	45	7.6
	H_2_O, 3 h, 90 °C, stirring	574	57	9.7
	KOH, 6 h, 50 °C, stirring	3834	383	64.4
	H_2_O, 9 h, 30 °C, ultrasonic bath	689	69	11.6
	H_2_O, 9 h, 50 °C, ultrasonic bath	715	72	12.0
	H_2_O, 9 h, 60 °C, ultrasonic bath	634	63	10.7

The results presented in Table 3 indicate that, under the selected conditions, extraction with 0.5 M KOH resulted in substantially higher carbon recovery than extraction with H_2_O, with efficiencies of approximately 37–76% and 4–12%, respectively. The highest recovery was obtained for KP WD under stirring with 0.5 M KOH at 90 °C for 6 h (approximately 76%), while high recoveries were also obtained for TEV under heated and stirred alkaline conditions (approximately 67–69%). Overall, these findings show that the use of 0.5 M KOH combined with elevated temperature was the most effective approach for organic carbon recovery from the investigated digestates, while additional stirring was beneficial under some conditions.

## 4 Conclusion

The dried solid fractions of three Lithuanian digestates contained substantial amounts of organic matter and plant nutrients, supporting their potential for further valorization. In the dried digestate samples prior to extraction, total nitrogen reached approximately 4.5%, organic carbon accounted for up to 59.5%, and the maximum P_2_O_5_ and K_2_O contents were 4.4 and 1.0%, respectively. Structural and thermal analyses revealed clear differences among the materials: ARB and TEV were predominantly amorphous, whereas KP contained distinct crystalline mineral phases. The main mass-loss stage amounted to approximately 63–72%.

Across the investigated conditions, the extractant type produced the largest numerical differences in TOC concentration, whereas the effects of temperature, extraction time, particle size, and extraction mode were condition- and digestate-dependent. Alkaline extraction was markedly more effective than aqueous extraction, producing TOC concentrations of up to approximately 1900 mg/L after 3 days, compared with less than 300 mg/L in water. The highest TOC concentration, 4495 mg/L, and the highest carbon recovery efficiency, approximately 76%, were obtained from KP WD using 0.5 M KOH at 90 °C under magnetic stirring for 6 h. Ultrasonic treatment produced the highest TOC concentration observed among the aqueous extracts, reaching 715 mg/L for KP CD at 50 °C after 9 h, whereas particle size had only a limited and condition-dependent influence.

Overall, the results show that digestates may serve not only as nutrient-containing materials but also as sources of recoverable soluble organic carbons. However, the extraction performance varied among the digestates, demonstrating that processing conditions should be selected according to their individual composition. The calculated recovery efficiencies describe the transfer of organic carbon into the liquid phase and should not be interpreted as evidence of carbon sequestration, greenhouse gas mitigation, or climate benefit. Moreover, the strongly alkaline pH and elevated electrical conductivity of the KOH extracts indicate that their direct agronomic application cannot be recommended without further treatment and evaluation. Further characterization of the recovered organic compounds, together with carbon stability assessments, soil incubation, pot or field experiments, and broader environmental evaluations, is required before their use in liquid fertilizers or soil amendments can be recommended.
